# Time-Series Generative Adversarial Network Approach of Deep Learning Improves Seizure Detection From the Human Thalamic SEEG

**DOI:** 10.3389/fneur.2022.755094

**Published:** 2022-02-16

**Authors:** Bhargava Ganti, Ganne Chaitanya, Ridhanya Sree Balamurugan, Nithin Nagaraj, Karthi Balasubramanian, Sandipan Pati

**Affiliations:** ^1^Department of Electronics and Communication Engineering, Amrita School of Engineering, Amrita Vishwa Vidyapeetham, Coimbatore, India; ^2^Texas Institute of Restorative Neurotechnologies, University of Texas Health Science Center, Houston, TX, United States; ^3^Department of Neurology, University of Alabama at Birmingham, Birmingham, AL, United States; ^4^School of Biotechnology, National Institute of Technology Calicut, Kozhikode, India; ^5^Consciousness Studies Programme, National Institute of Advanced Studies, Bengaluru, India

**Keywords:** temporal lobe epilepsy, thalamus, Bidirectional Long-Short Term Memory (Bi-LSTM), seizure detection algorithm, Generative Adversarial Network (GAN)

## Abstract

Seizure detection algorithms are often optimized to detect seizures from the epileptogenic cortex. However, in non-localizable epilepsies, the thalamus is frequently targeted for neuromodulation. Developing a reliable seizure detection algorithm from thalamic SEEG may facilitate the translation of closed-loop neuromodulation. Deep learning algorithms promise reliable seizure detectors, but the major impediment is the lack of larger samples of curated ictal thalamic SEEG needed for training classifiers. We aimed to investigate if synthetic data generated by temporal Generative Adversarial Networks (TGAN) can inflate the sample size to improve the performance of a deep learning classifier of ictal and interictal states from limited samples of thalamic SEEG. Thalamic SEEG from 13 patients (84 seizures) was obtained during stereo EEG evaluation for epilepsy surgery. Overall, TGAN generated synthetic data augmented the performance of the bidirectional Long-Short Term Memory (BiLSTM) performance in classifying thalamic ictal and baseline states. Adding synthetic data improved the accuracy of the detection model by 18.5%. Importantly, this approach can be applied to classify electrographic seizure onset patterns or develop patient-specific seizure detectors from implanted neuromodulation devices.

## Introduction

Despite significant advances in diagnostic and therapeutic technologies, over 30 million people worldwide have drug-resistant epilepsy (DRE) (1). Increased seizure burden plays a central role in morbidity and mortality, thereby emphasizing the need for seizure preventative therapies (2). Surgical resection of the seizure focus may yield seizure freedom and remains the first line of treatment in DRE. However, in many patients, resection or ablation is not an option if the seizure foci are widespread involving multiple regions or are non-localizable (3, 4). Neuromodulation of the epileptogenic circuit remotely *via* a central hub like the thalami is often the treatment of choice in this cohort (5). Accurate and timely detection of seizures is clinically necessary for the development of feedback “responsive” therapy and monitoring seizure counts for therapy adjustment. In recent years, the medical community has widely adopted machine learning approaches to develop seizure detection algorithms. Various linear and non-linear features are extracted and have been used for seizure detection and prediction (6–12). However, these seizure detection algorithms have been optimized from electrophysiological signals obtained from the seizure focus. Machine learning algorithms to detect seizures from outside the seizure focus are still in their nascency (13).

There are multiple challenges in developing seizure detectors from regions like the thalamic subnuclei, i.e., (a) the thalami have lower power spectra, and the spectral contents are significantly different from the epileptogenic cortex during interictal and seizure substages (14), (b) the thalami are not routinely implanted during surgical evaluation, and hence electrophysiological recordings during seizures are scarce. Thus, the sample size is small and often inadequate for data-intensive deep learning models, and (c) Chronic local field potentials (LFPs) can be recorded from the thalami in patients with sensing-enabled deep brain stimulators (DBS) and can potentially be the solution to inadequate data. However, establishing the accuracy of detecting seizures in the ambulatory setting is challenging. In the proposed work, we overcome the inadequate sample size by applying a novel deep learning approach for detecting seizures from LFPs recorded directly from the human thalamic subnuclei.

Several deep learning algorithms have been proposed for automatic seizure detection. These include artificial neural networks, convolution, and deep convolution-based seizure detection systems. Amongst them, a widely popular and high-performing method for seizure classification using EEG is the use of temporal models such as recurrent neural network (RNN) and its variants, including Long-Short Term Memory (15)(LSTM) Bidirectional LSTM (BiLSTM), Gated Recurrent Unit (GRU) (16), and Generative Adversarial Networks (GAN). LSTMs are known for their excellence in learning patterns from temporal information while preserving dependencies in very long-time sequences. However, these temporal models (RNN, LSTM, GRU) are first trained in an adequately powered sample to learn the inherent temporal dependencies of the EEG signal that accurately represent the features of a seizure. In the present study, we apply the time-GAN method with the novel goal of detecting temporal lobe seizures from a limited number of the LFPs recorded from the human thalami. We hypothesize that the performance of a deep learning algorithm classifying seizures from the interictal state can be significantly improved by adding synthetic data using the GAN approach.

## Methods

### Study Participants and Ethics

Patients diagnosed with drug-refractory temporal lobe epilepsy (TLE) who underwent stereoelectroencephalography (SEEG) for localization of seizure focus were included in the study. The indication for SEEG was clinically necessary and determined in a multidisciplinary patient management conference. Within this cohort, consenting adults who had thalamic implantation for research were included in the present analysis. The multi-step consenting and evaluation process has been described in detail in our previous studies (13, 14, 17). The electrophysiological sampling of the thalamic subnuclei was performed under the supervision of the IRB, and all patients provided written informed consent. To mitigate the risk associated with implanting an additional depth electrode for research sampling of the thalamus, we modified the trajectory of a clinically indicated depth electrode sampling the operculum-insula to track medially for recording from the thalamus. Clinician-identified seizures were documented for all patients, and the SEEG data was clipped and parsed for analysis. Ictal (*N* = 84 from 13 patients) and baseline interictal data (Length: 550 s prior to seizure) were obtained. The demographic details of the study participants are detailed in Table 1.

**Table 1:** Demographic details of the study participants.

**Demographics**	***N* = 13**
Age (years)	42.8 ± 11.9
Gender (M:F)	6:7
**Details of recording:**	
Number of contacts	2,205 (R: 1,328, L: 877)
Thalamic implant laterality (R:L)	8:3
Thalamic target nucleus (Anterior: Central)	8:3
**Disease burden measures:**	
Age at Onset (years)	28.8 ± 16.2
Duration of Epilepsy (years)	14.3 ± 16.3
Frequency of focal seizures (/month—median and range)	4 (range: 1–48)
H/o FBTCS (Present: Absent)	6:7
MRI (Abnormal: Normal)	7:6

*M, male; F, female; FBTCS, focal to bilateral tonic-clonic seizures; MRI, magnetic resonance imaging*.

### SEEG Recording

All SEEG implantation procedures were performed using robotic assistance (ROSA device, MedTech, Syracuse, NY) (12–16 contacts per depth electrode, 2 mm contact length, 0.8 mm contact diameter, 1.5 mm intercontact distance, PMT® Corporation, Chanhassen, MN). Once implanted, the patients were monitored over 4–12 days in the epilepsy monitoring unit (EMU). SEED data as recorded using Natus Quantum (Natus Medical Incorporated, Pleasanton, CA, sampling rate 2,048 Hz). Signals were referenced to a common extracranial electrode placed posteriorly in the occiput near the hairline.

### Accurate Anatomical Localization of SEEG Depth Electrodes

The details of the accuracy of the implantation strategy have been reported in our previous study (17). Here we highlight the main steps to localize the SEEG electrodes to the various cortical regions and the thalamic subnculei. The post-implant CT-scan was coregistered to preimplant MRI using Advanced Normalization Tools (ANTs) and refined registration of deep structures was performed using brain shift correction to improve the registration of subcortical structures using Lead-DBS v2 software (18). Both the images were normalized to ICBM 2009b NLIN asymmetric space using the symmetric diffeomorphic image registration. Following this, the localization of the thalamic contacts was performed in Lead-DBS, while the cortical channels were performed in iElecetrodes (19). Thalamic contacts were registered to Morel's thalamus atlas, while cortical contacts were localized using AAL2 atlas (20).

### Identification of Interictal Epochs and Seizures in the Seizure Onset Zone and Thalamus

The time of the seizure onsets and offsets was annotated by a board-certified epileptologist (SP). Seizure onset in the cortex was marked as “unequivocal EEG onset” (UEO) at the earliest occurrence of rhythmic or repetitive spikes that was distinct from the background activity. SEEG segments were clipped to include 10 min before this UEO and 10 min after seizure termination. Four different clinical seizure types were included for analysis: focal aware seizures (FAS), focal impaired awareness seizures (FIAS), and focal to bilateral tonic clonic seizures (FBTCS) (21). Epochs of “interictal state” (28 epochs with each epoch lasting 9 min) were visually screened and identified from the non-seizure segment of the SEEG that was at least 1 h preceding seizure. Our previous study showed that the interictal spikes in baseline data need not be actively removed for classifying ictal states from baseline states (13). Secondly, interictal spikes will be present while training real-time data, e.g., line length detection in responsive neuromodulation systems (RNS) (22). Hence for translational purposes, no effort was made to exclude epileptiform spikes in the baseline epochs. Supplementary Material shows the details of the ictal and baseline data.

### Deep Learning Architectures

**Figure 1:**
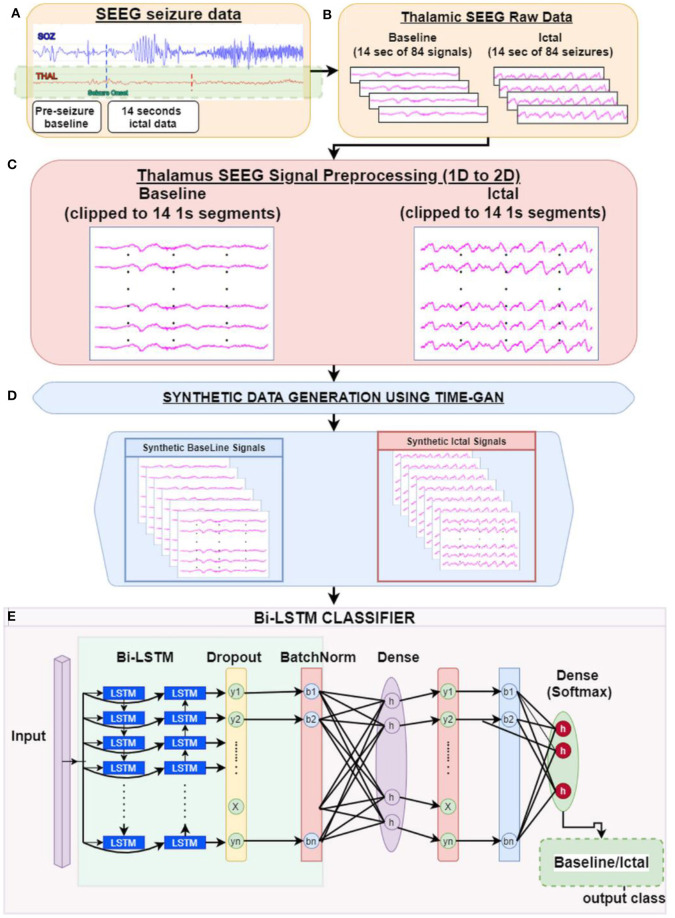
Study pipeline: **(A)** Clinician identified seizure onset timings in the hippocampal-amygdalar complex were determined. We then clipped thalamic EEG segments into epochs of baseline and seizure onset. **(B)** Each epoch consisted of 14 s of raw thalamic EEG segments **(C)**. As an initial step, each 14-s 1D signal epoch was fragmented into 1-s segments to generate a 2D matrix of time × signal (sampling rate: 2,048 samples/s) **(D)**. The data was then submitted to the TGAN system to generate synthetic data at the individual subject level. TGAN is expected to generate synthetic data that mimics original data and augment the sample size required for deep learning. **(E)** Two separate bidirectional long short-term memory (BiLSTM) models were tested independently on original and synthetic data.

The input to the BiLSTM classifier was the interictal baseline and ictal thalamic EEG data. The baseline and the ictal data from the 84 seizures (13 subjects) were initially grouped by the subject identification. Since the length of ictal data was variable, we chose the length of the shortest seizure for any given subject (i.e., 14 s) as the length of the analyzable data. To avoid a discrepancy in the length of data between ictal and baseline segments, we chose a similar 14 s length of SEEG data from the initial segment of the baseline data. The input to the BiLSTM classifier is a 2D array of data. Hence, the 14 s of the data were then clipped into multiple 1 s epochs and rearranged into a two-dimensional array of 14 × 2,048 samples (Figures 1A–C).

**Figure 2:**
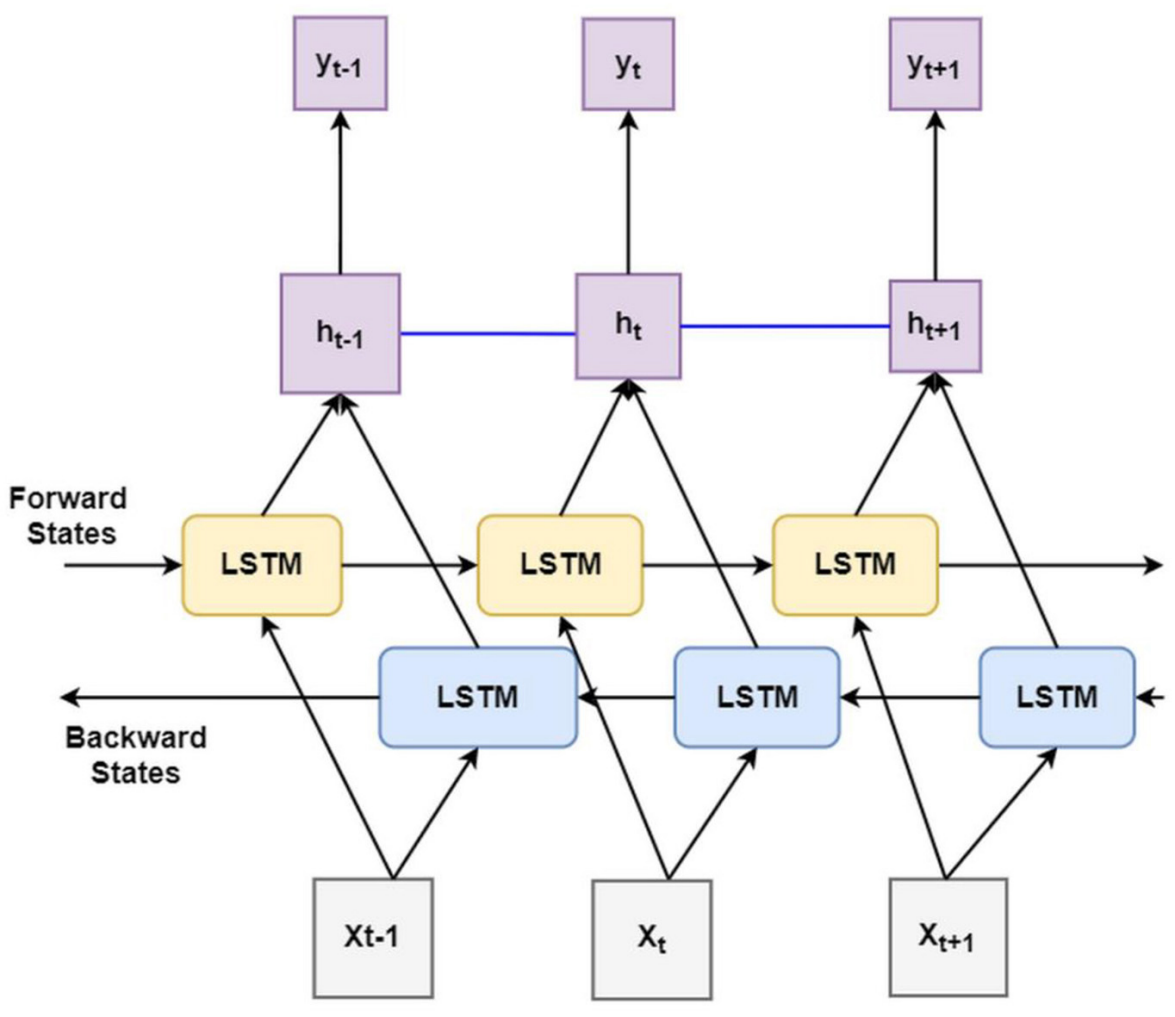
Block diagram of bidirectional long short-term memory learning (BiLSTM): A BiLSTM, is a serial sequence learning model that consists of two LSTMs operating in two directions effectively increase the amount of information available to train and test the network. The first LSTM inputs in data in a forward direction, and the second LSTM in a backwards direction. This improved the context available to the learning algorithm helping it to learn the sequence of the time series data, i.e., what data immediately follows (X_t+1_) and precedes (X_t−1_) the events of interest such as the seizure (X).

Principally, the LSTM network only obtains information from the previous input observations but cannot use that information for future input observations. However, the BiLSTM model, composed of two independent LSTM networks, can transmit information bi-directionally and increase the learning ability of the system output (Figure 2) (23). Sixty three seizures from 11 subjects (were used to train the classifier differentiating ictal from baseline. Subsequently, 21 seizures collected from 2 patients were used for testing the model. Each BiLSTM classification model consisted of 64 units of LSTMs in the encoding layer and a kernel regularizer of 12 followed by a drop-out layer with a drop-out ratio of 0.25 and a batch normalization layer. This was followed by a dense layer of 64 units with rectified layer unit (ReLU) activation function (24). For the final output, a dense layer with SoftMax activation function of two units was used for the binary classification of baseline interictal and ictal states.

### Generation of Synthetic Data With GAN

**Figure 3:**
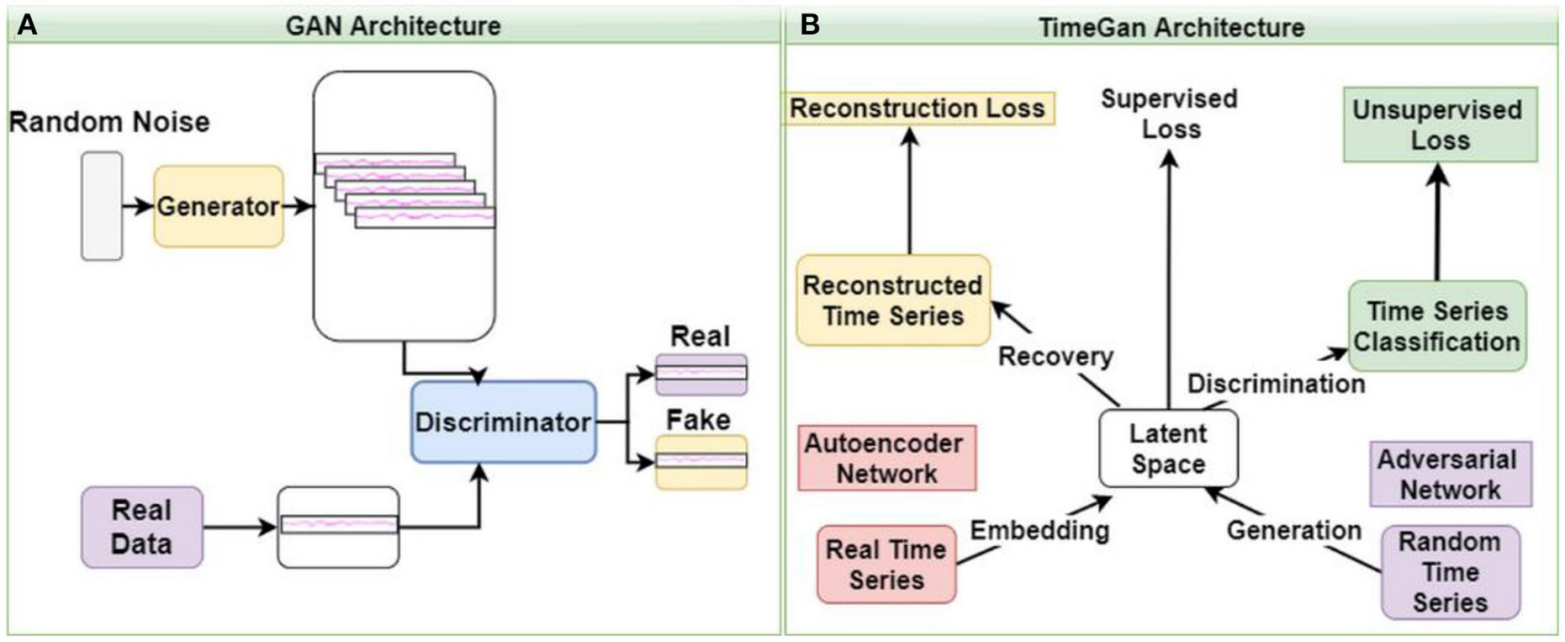
The architecture of GAN **(A)** and time GAN **(B)**. **(A)** Generative Adversarial Networks (GAN) is an unsupervised learning system that involves discovering and learning the patterns in input data to generate a new set of synthetic data that mimics the original dataset. **(B)** Time-series Generative Adversarial Networks (Time-GAN) combines the flexibility of the unsupervised paradigm with the control by incorporating supervised training.

GANs learn and generate synthetic data by preserving the data distributions. For a generation of sequential data, the temporal dynamics need to be preserved. Yoon et al. (25) proposed the concept of time-series GAN (TGAN) that was able to capture not only the distribution of data at each instant but also the presence of various features across time (Figures 3A,B). TGAN differs from other GAN architectures in two ways. (a) By introducing an embedding network, it reduces the dimension of the adversarial learning space, and (b) uses supervised adversarial loss, unlike GAN, where unsupervised methods are used. In our analysis, TGAN was used to generate synthetic data that was 10 times the original data. The data (ictal and baseline) were fed into the TGAN model to produce the augmented data (Figure 1D).

### Validation of Synthetic Data

The second level BiLSTM analysis classifies ictal and baseline states based on the synthetic TGAN data (Figure 1E). Hence it was necessary to validate the similarity of the synthetic data with the original data. The validation was quantified using the Train-Synthetic-Test-Real method (TSTR), where a logistic regression classifier model with a single layer gated recurring units (GRU) with 12 units was used for training both the original and the synthetic data. Each model was trained separately with 75% of the original and the synthetic data, respectively. The testing of both the models is done with the remaining 25% of only the original data set (25). This allowed us to estimate individual seizure level and subject level coefficient of determination (*R*^2^) values and the percentage difference between original and synthetic data (Table 2). Finally, a *t*-distributed stochastic neighbor embedding (*t*-SNE) analysis was performed to visualize if the ictal and baseline data could be better segregated using the original or the synthetic data. T-SNEs were generated in MATLAB using the “*exact*” algorithm, with Mahalanobis distance, the perplexity of 50, and PCA dimensions of 3.

**Table 2:** Coefficient of determination (R2) and mean absolute error (MAE) values obtained from regression models comparing original and synthetic data for the baseline and ictal data.

**S_ID**	**#Seizures**	**Baseline**					
**Validation**		****R** ^**2**^**			**MAE**		
		**Original**	**Synthetic**	**% Difference**	**Original**	**Synthetic**	**% Difference**
7	10	0.468	0.469	0	3.219	3.219	0
8	7	0.367	0.370	1	9.220	9.146	1
9	9	0.260	0.268	3	4.876	4.877	0
10	7	0.182	0.180	1	20.189	20.155	0
14	8	0.285	0.275	4	4.349	4.348	0
15	6	0.850	0.880	3	32.485	32.350	0
16	11	0.910	0.920	1	8.976	8.976	0
17	3	0.253	0.250	1	4.348	4.368	0
18	3	0.413	0.420	2	7.171	7.170	0
19	5	0.380	0.380	0	3.104	3.104	0
20	4	0.400	0.420	5	12.628	12.608	0
21	6	0.680	0.640	6	12.979	12.881	1
22	5	0.854	0.866	1	10.977	10.977	0
	**Group**	0.484 ± 0.251	0.487 ± 0.256	2 ± 1%	10.34 ± 8.25	10.32 ± 8.21	0.1 ± 0.3%
		**Ictal**					
7	10	0.868	0.869	0	2.958	2.957	0
8	7	0.456	0.470	3	14.710	14.710	0
9	9	0.380	0.370	3	4.828	4.828	0
10	7	0.218	0.210	4	23.593	23.602	0
14	8	0.360	0.365	1	6.533	6.533	0
15	6	0.420	0.426	1	59.375	59.376	0
16	11	0.910	0.910	0	5.825	5.825	0
17	3	0.340	0.335	1	5.215	5.215	0
18	3	0.413	0.420	2	8.114	8.144	0
19	5	0.278	0.269	3	5.617	5.636	0
20	4	0.400	0.400	0	3.729	3.729	0
21	6	0.600	0.600	0	21.081	21.082	0
22	5	0.750	0.760	1	6.807	6.661	2
	**Group**	0.491 ± 0.221	0.492 ± 0.224	1 ± 1	12.95 ± 15.42	12.94 ± 15.42	0.1 ± 0.5

*S_ID, Subject Identification number; #, number of; R^2^, coefficient of determination; MAE, mean absolute error; % difference, absolute percentage change in original and synthetic*.

### Performance of BiLSTM on Original vs. Synthetic Data

To estimate the performance of the BiLSTM following metrics were computed: sensitivity (Sn or recall), specificity, accuracy (for training, validation, and testing data), positive predictive value (PPV or precision), F1-score, and area under the curve (AUC). The difference in the performance of the BiLSTM classifiers for original and synthetic data was visualized using ROC (receiver operating characteristic) curve by testing the relationship between sensitivity and 1-specificity.

### Implementation Details

The BiLSTM and TGAN models were tested in Python, and t-SNE analysis was performed in MATLAB. We utilized Keras (26), scikit-learn, an open-source Python API that takes into account the neural organization structures based on top of TensorFlow, to construct all learning models.

## Results

### Safety and Localization of Thalamic Electrodes

**Figure 4:**
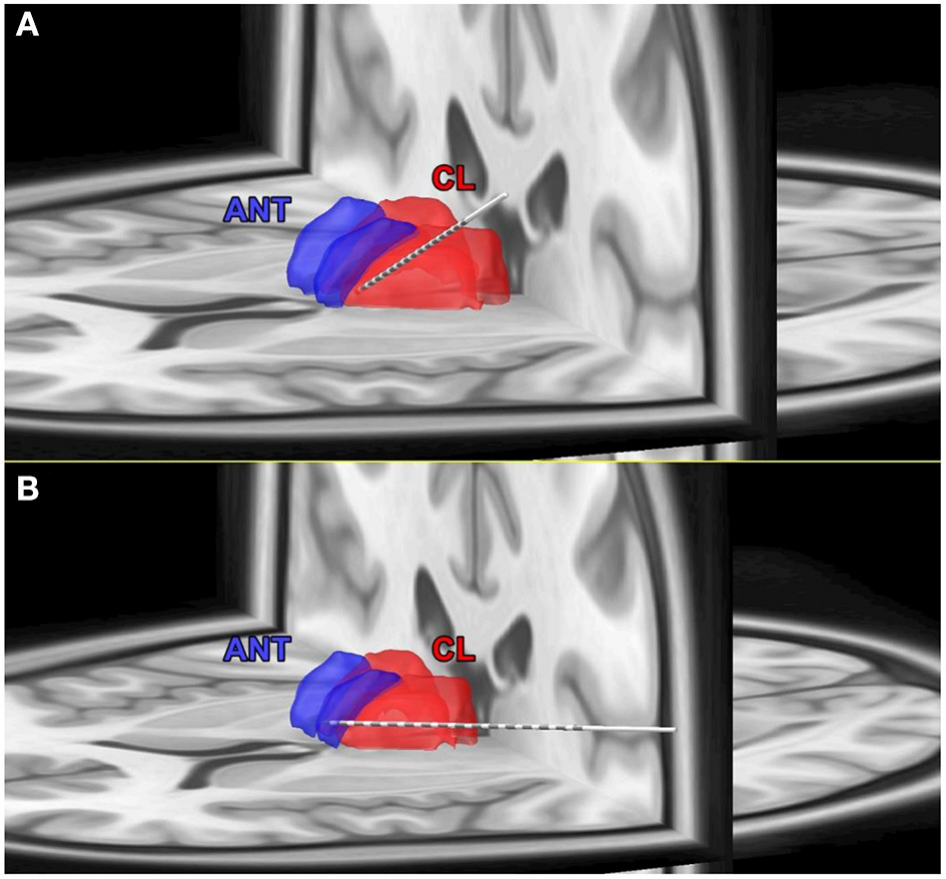
**(A)** Coregistration of post-implant CT scan on pre-implant MRI and Morel's thalamic atlas to determine the exact localization of thalamic targets. **(A)** An example of the electrode localized to the central thalamic nucleus (CL). **(B)** An example of the electrode localized to the anterior thalamic nucleus (ANT).

Thirteen subjects were included in the study, with 10 had electrodes localized to the anterior nucleus of the thalamus (ANT) and 3 in the centrolateral thalamic nuclei (Table 1, Figure 4). CT brain (post-implant and post-explant) did not show any thalamic hemorrhage. Eight subjects were implanted on the right side and three on the left side.

### Clinico-Demographic Details of Subjects

Table 1 summarizes the clinic-demographic details of the subjects included in this study. A total of 84 seizures from 13 subjects were analyzed. The seizure onset zone was determined based on the clinical consensus among the epileptologists during the epilepsy surgical conference. The identified seizure focus was: medial temporal (4 subjects), mesial + temporal pole onset (3 subjects), temporal plus (5 subjects), with the plus representing additional seizure foci (orbitofrontal or insula or suprasylvian operculum) (27). The seizure types were: ES (19), FAS (24), FIAS (28), and FBTCS (6).

### TGAN Augmented Synthetic Data Were Comparable to the Original SEEG Data

The TGAN generated synthetic data was similar to the original SEEG data. At baseline, there was no difference between the mean coefficient of determination (*R*^2^) of the original and synthetic data (original: 0.484 ± 0.251, synthetic: 0.487 ± 0.256, *t* = −0.6, *p* = 0.27). Similarly, there was no difference in the mean absolute error (MAE) of original and synthetic data (original: 10.34 ± 8.25, synthetic: 10.32 ± 8.21, *t* = 0.008, *p* = 0.49). Similarly, the TGAN augmented data synthesized during the ictal period did not differ from the original data in *R*^2^ (original: 0.491 ± 0.221, synthetic: 0.492 ± 0.224, *t* = −0.0097, *p* = 0.49) and the MAE (original: 12.95 ± 15.42, synthetic: 12.94 ± 15.42, *t* = 0.001, *p* = 0.49).

### TGAN Augmented Synthetic Data Enhanced the Performance of the BiLSTM Classifier

**Figure 5:**
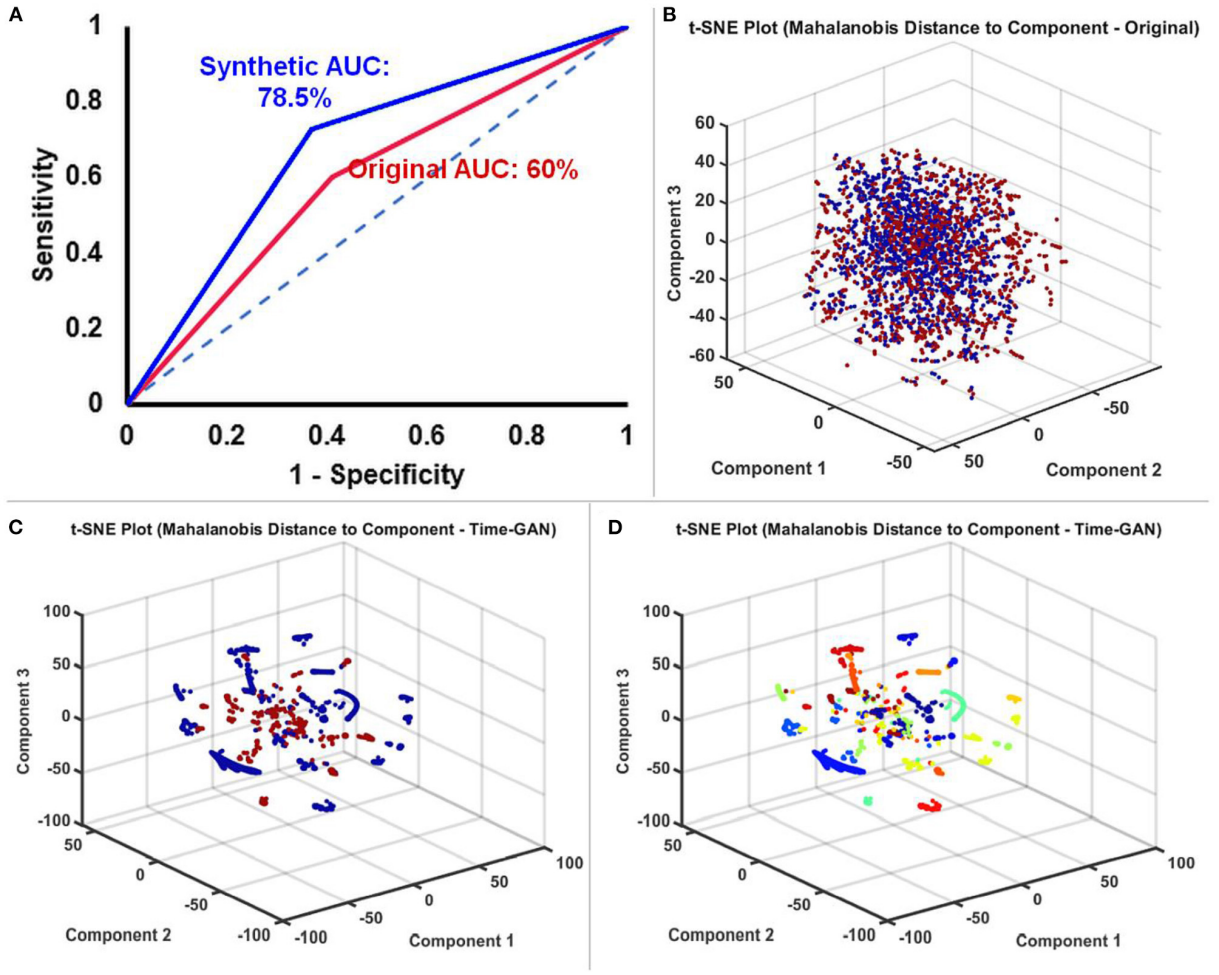
**(A)** Receiver operator characteristic (ROC) curves comparing the performance of BiLSTM models trained with original and TGAN augmented synthetic data. **(B)** T-SNE plot of the original SEEG data showing the baseline (red) and ictal (blue) data. **(C)** T-SNE plot of the TGAN augmented synthetic data shows a clear distinction between the two groups (baseline in red and ictal in blue). **(D)** A t-SNE indexed by the subject ID showed that TGAN amplifies the ictal data specific to each patient that is distinctly different from their comparable baselines (the different colors are indicative of the data different 13 different subjects). In conjunction with C, we understand that data is not only classified based on ictal and interictal data, but also distinctly clustered based on individual subjects' data.

We constructed ROC curves to determine the performance of the BiLSTM on original, and TGAN augmented synthetic data. The classification of the ictal from the baseline data was superior with the synthetic TGAN augmented data compared to the original data (original: AUC: 60% and synthetic: 78.5%, Figure 5A). This improvement in the performance of the BiLSTM models could be better visualized using three component t-SNE plots (Figures 5B–D). T-SNE of original data failed to parse the ictal and baseline clusters separately (Figure 5B), while T-SNE performed on the TGAN augmented synthetic data with the same parameters, demonstrated a clear separation into ictal and the baseline clusters (Figure 5C). We initially noted that the ictal clusters were further separated in space into multiple clusters. A t-SNE indexed by the subject ID showed that TGAN amplifies the ictal data specific to each patient that is distinctly different from their comparable baselines (Figure 5D). The result suggests that the patient-specific electrographic seizure onset patterns were retained in the TGAN augmented data (Figure 5D).

**Figure 6:**
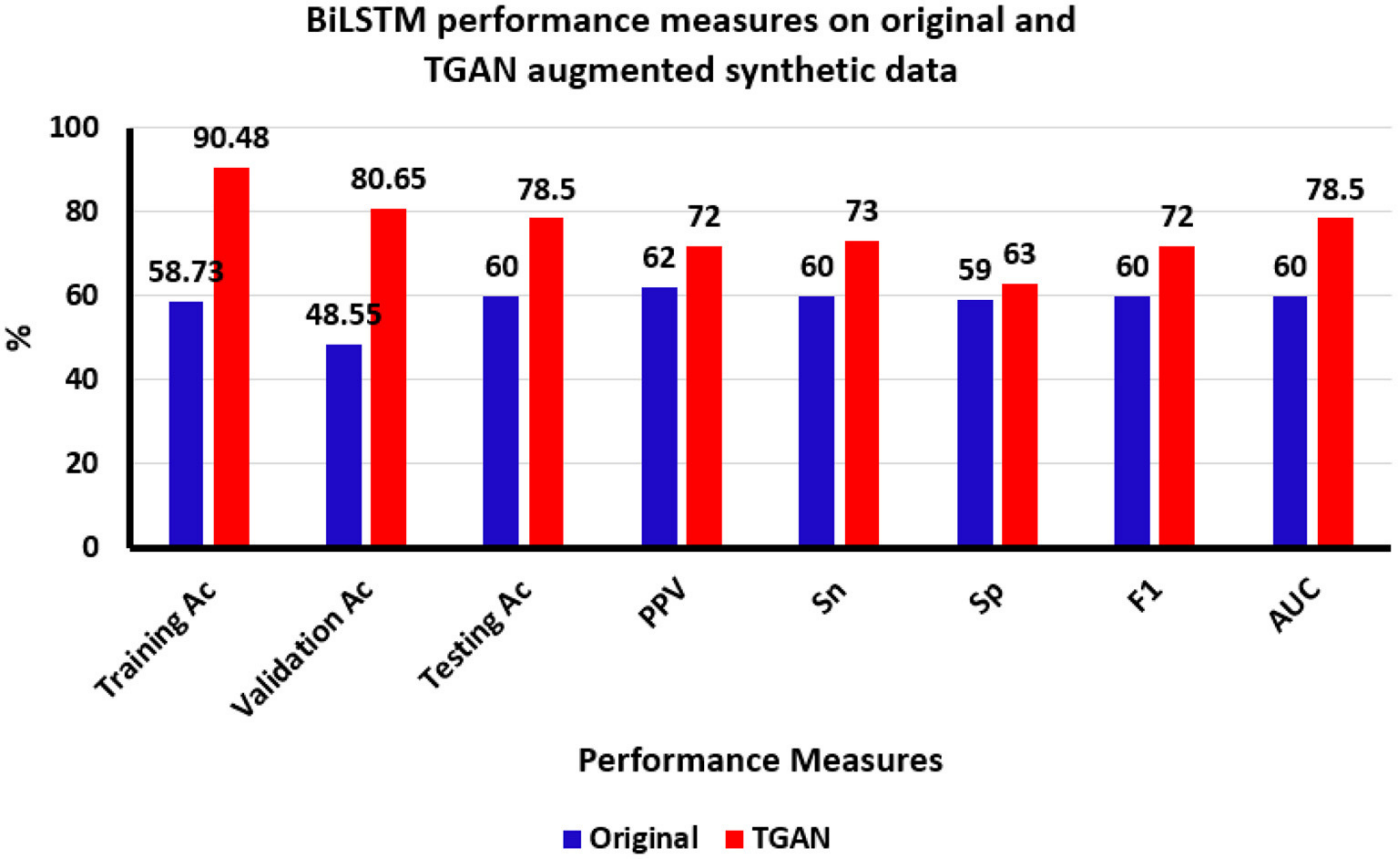
Bi-directional Long Short-Term memory (BiLSTM) classification results using original and time generative adversarial networks (Time-GAN) generated synthetic data. Ac, accuracy; PPV, positive predictive value, Sn, sensitivity; Sp, specificity; F1, F-score; AUC, area under the curve, %, percentage.

Overall, the performance of the BiLSTM in classifying ictal and baseline states from thalamic SEEG data was enhanced by the use of TGAN generated synthetic data over the original data. The accuracy of the training data improved by 31.75%, the validation data improved by 32.1%, and finally, the testing data improved by 18.5%. The sensitivity and PPV of the BiLSTM classifier on improved by 13 and 10% on the testing data (Figure 6).

## Discussions

Currently the only clinically available neuromodulation system that is based on a close loop system approved by the United States Food and Drug Administration (FDA) is the Responsive neurostimulation. To date this device has been extensively used to target neuromodulation in the cortical regions. This device uses amplitude threshold and line-length as the main seizure detection algorithms. There have been anecdotal reports of implanting the human thalamus with RNS, where the seizures were still detected in the cortex but the stimulation was performed in the ANT. There has been growing literature that thalamus is involved early in focal seizures, particularly in TLE. Some studies have also tried to detect seizures from human thalamus. This detection of seizures from the human thalamus and understanding the pattern of involvement of thalamus on focal seizures is of utmost importance while developing closed-loop DBS systems. To date, it has been shown that ANT DBS (open loop) has had great success in patients with drug resistant epilepsy particularly those patients who are negative for a lesion on the MRI, with a median seizure frequency reduction of 75% at 7 years of therapy.

Newer sensing-enabled DBS systems have been approved by the FDA since 2018 in the practice of epilepsy and since 2002 for Parkinson's disease. These devices offer closed loop sensing and diary functions (record of events) to monitor symptoms and tailor therapeutic stimulation. A recent study has shown that closed loop neurostimulation within the human thalamus has shown a ≥50% reduction in seizure frequency with no adverse effects on mood, memory or behavior. With the advent of such sensing-enabled closed loop systems, there is a clinical need to develop seizure detection algorithms from the thalamic SEEG and not just from the cortical seizure onset zone. One of the most critical steps in enabling sensing, is to develop patient-specific detection based on individual subject's thalamic seizure patterns. Often, the data obtained from a single subject is limited and hence the translation of deep learning approaches has been hindered by the lack of larger samples of curated ictal thalamic SEEG needed for training these classifiers. Here, we demonstrate the utility of generating synthetic data using GAN that can augment the sample size and improve the performance of BiLSTM. Importantly, this approach can be applied to classify electrographic seizure onset patterns or develop patient-specific seizure detectors from implanted neuromodulation devices. In summary, we found that Time-GAN helps generate synthetic time series that resemble the original data, with a very small mean absolute error rate of 0.1 ± 0.5% between the original and the augmented data. In fact, when this time-GAN augmented data was used in BiLSTM classifier to detect the ictal state, we noticed that the accuracy of the classifier improved by 18.5%, sensitivity by 13% and PPV by 10% when compared to classifying using the original data. Though marginal, such an improvement is promising and further refinement of such models are required to optimize seizure detection in the thalamus.

### Performance of Deep Learning Algorithms for Seizure Detection

Table 3 summarizes the performance of deep learning algorithms in detecting seizures from electrophysiological signals recorded from the scalp and intracortical regions (LFPs). To date, deep learning algorithms to detect seizures were applied to EEG obtained from the cortical areas that participate in seizure generation. Our study is distinct and the first of its kind to perform deep learning detection on EEG recordings from a brain region that is remote to the seizure focus.

**Table 3:** Summary of prior studies evaluating deep learning for seizure detection.

**References**	**Algorithm**	**No. of classes**	**No. of patients:No. of seizures**	**Accuracy (%)**	**Data type**
Tsiouris et al. (29)	LSTM	2	23:198	99.8	ICEEG
Ullah et al. (30)	CNN	2	5:100	99.8	Scalp and ICEEG
Abdelhameed et al. (31)	Deep LSTM	5	5:100	100	Scalp and ICEEG
Avcu et al. (32)	Deep CNN	2	29:120	93.3	ICEEG
San-segundo et al. (33)	Deep CNN	3	5, 500:3,750, 11,500	95.7	ICEEG
Lu et al. (34)	Deep CNN	3	5, 500:3,750, 11,500	91.8	Scalp
Asif et al. (35)	SeizureNet	2	500:11,500	94.0	Scalp
Yao et al. (28)	BiLSTM	2	23:665	84.55	Scalp
Hu et al. (36)	BiLSTM	2	23:665	93.61	Scalp
Yan et al. (37)	CNN	2	679:177	98	ICEEG

*ICEEG, intracranial EEG; LSTM, long short term memory; BiLSTM, bidirectional long short-term memory; CNN, Convolutional Neural Network*.

As expected, the performance of these classifiers was higher with biosignals obtained directly from the cortex than from the scalp which is likely to be closer to the seizure focus. In those studies, the sample size for deep learning consisted of over 100 ictal EEG data. The main motivation of this study is to highlight how data augmentation techniques can improve the accuracy of the classifier, albeit a lower overall performance of our classifier in comparison to other studies. Even when data is smaller (84 seizures), we can use data-augmentation methods to enhance the performance of the classifier (accuracy improved by 18% in our current study) and improve the detection performed in subcortical neuromodulatory targets such as the thalamus, which are distant and outside the seizure cortex. Wei et al. (38) were among the pioneering teams in demonstrating improved seizure detection in scalp EEG with GAN models. They used the Wasserstein Generative Adversarial Nets (WGANs) combined with a convolutional neural network (CNN) to demonstrate a 3% improvement in accuracy (81.5–84.4%) and a near 2% improvement in the sensitivity (70.68–72.11%). In our study, the accuracy and sensitivity improved by 18.5 and 13%, respectively. Zhao et al. (39), in their model with a 1D-CNN with data-augmentation on data obtained from intracranial EEG data that was close to seizure focus, achieve an improvement of only 3% (accuracy of 89.28% compared to 86.89 with a support vector machine). They proposed a data augmentation method which leverages feature correlations in the transformed domain rather than in the original domain where time-domain data is converted to the frequency domain by discrete cosine transform (DCT), and new artificial data is generated by combining different frequency bands from different data, and converted back to time-domain data. Overall, these studies and ours, point to the promising future of using data augmentation techniques for better seizure detection to improve therapeutic stimulation.

### Augmented Subject-Specific Classification With Temporal GAN

GANs, are deep-learning algorithms where two competing networks, namely the generator and the discriminator, compete against each other until the generator generates artificial data of high quality. According to Goodfellow et al. (40), “the generative models are analogous to a team of counterfeiters trying to produce fake currency without being detected. The discriminative model is analogous to the police trying to detect fake currency. The competition between the generator and discriminator drives improvement until the counterfeiters are indistinguishable from the genuine currency.” Thus, GAN has been used to classify interictal spikes and in EEG-based brain-computer interfaces. Our result supports the use of GAN to produce synthetic data to augment the performance by 18% as compared to using only the original data. The ability to classify seizures from limited samples of unprocessed LFP signals may provide a clinical advantage in neuromodulation devices where efficient processing at a lower computational expense is desired.

### Study Limitations

There is a proof-of-concept study evaluating the use of synthetic data in augmenting sample size for deep learning. The study needs to be extended to a larger cohort with thalamic recordings of seizures and interictal baseline. One major challenge of the temporal GAN model is that it is computationally intense and consumes time to learn or converge to local minima and hence slows the training process. Another limitation of GAN is that the presence of discontinuous (e.g., ECoGs obtained from clinical neuromodulation devices) data may synthesize incorrect data. In our study, the duration of the data used for time-GAN analysis was 14 s based on the shortest duration of the ictal event from our cohort and in the future the results need to be optimized to individual patients, in whom the seizure durations are likely to vary significantly. This will also have a bearing on minimizing the detection latencies in the future models. Such sophisticated models will help build closed-loop neuromodulation strategies where early seizure detection can be used to pace the brain to abort seizures. Regarding the size of our data-set, we used 63 seizures from 11 patients' data for training and 21 seizures from 2 patients's data for testing the BiLSTM and Time-GAN models. While our study did show a reasonable improvement in the accuracy of the BiLSTM models and can be used as a proof of concept, in the future it is essential to validate this using random sampling of the patients' data and at individual subject level to emphasize and validate its clinical use. Also, cross validation across all patients' data would further strengthen the validity of the model. Since GAN is time consuming and resource demanding, our purpose was not to run it on all subjects but show how even in few subjects an improved accuracy can be obtained. Another limitation is that we did not determine the exact cause of improved accuracy and test the fidelity, diversity, and generalization of the data augmentation method, i.e., TGAN. These measures help determine the point at which the generative model surpasses and fools the discriminative network. Once the TGAN augmented data robustly mimics the real data, the TGAN-output is then used as the input in BiLSTM models improve accuracy. TGANs supersede BiLSTMs at finding a better low dimensional representation and hence may contribute to improved accuracy. In our study, we were interested in showing if the TGAN is able to produce synthetic data effectively from available limited samples and whether that use of the synthetic data shows elevated performance as compared to the using the original data only and a more detailed validation of TGAN was to voluminous for this study.

## Conclusion

The ability to detect seizures from the thalamus- a structure remote to the seizure focus is clinically necessary for monitoring seizure burden in drug-resistant epilepsies where seizure foci are non-localizable. In this study, we demonstrate the use of synthetic data to augment sample size and improve deep learning performance in detecting seizures from the human thalamic SEEG. The proposed framework should be extended to a larger cohort of patients with thalamic DBS in multifocal epilepsies.

## Data Availability Statement

The datasets presented in this article are not readily available because the data is controlled by IRB. Requests to access the datasets should be directed to patilabuab@gmail.com.

## Ethics Statement

The studies involving human participants were reviewed and approved by University of Alabama Birmingham Medical Center. The patients/participants provided their written informed consent to participate in this study.

## Author Contributions

BG and GC analyzed, interpreted, and helped in writing the mansucript. KB supervised data analysis, interpretation, and revised the mansucript. RB and NN provided analytical tools and helped in analysis of the data. SP obtained IRB approval, designed the study, interpreted the data, and wrote and revised the manuscript. All authors approved the final version of the manuscript.

## Funding

SP would like to acknowledge the USA National Science Foundation Grant (NSF RII-2 FEC OIA-1632891) that provided protected time for research.

## Conflict of Interest

The authors declare that the research was conducted in the absence of any commercial or financial relationships that could be construed as a potential conflict of interest.

## Publisher's Note

All claims expressed in this article are solely those of the authors and do not necessarily represent those of their affiliated organizations, or those of the publisher, the editors and the reviewers. Any product that may be evaluated in this article, or claim that may be made by its manufacturer, is not guaranteed or endorsed by the publisher.
