# Supplementary material for: Time-Series Generative Adversarial Network Approach of Deep Learning Improves Seizure Detection From the Human Thalamic SEEG
Source: Front Neurol. 2022 Feb 16;13:755094. doi: 10.3389/fneur.2022.755094 (PMC8889931; doi:10.3389/fneur.2022.755094)
Supplement: Supplementary file 1 [file Data_Sheet_1.PDF]

Supplementary Material:

<u>Table of Contents:</u>

LSTM:

LSTM (39) is a special version of RNN which surpasses the limitations of gradient vanishing by replacing the hidden layers with memory cells and gated mechanisms. The basic architecture of the LSTM model is shown in Figure2 and is composed of three gates namely input gate, forget and output gate respectively. The input gate determines whether to update the current state of the LSTM unit with an input sequence. The forget state is designed to retain or discard the previous LSTM unit selectively. The output gate determines whether the current hidden state is passed to the next iteration and also controls the output of information.

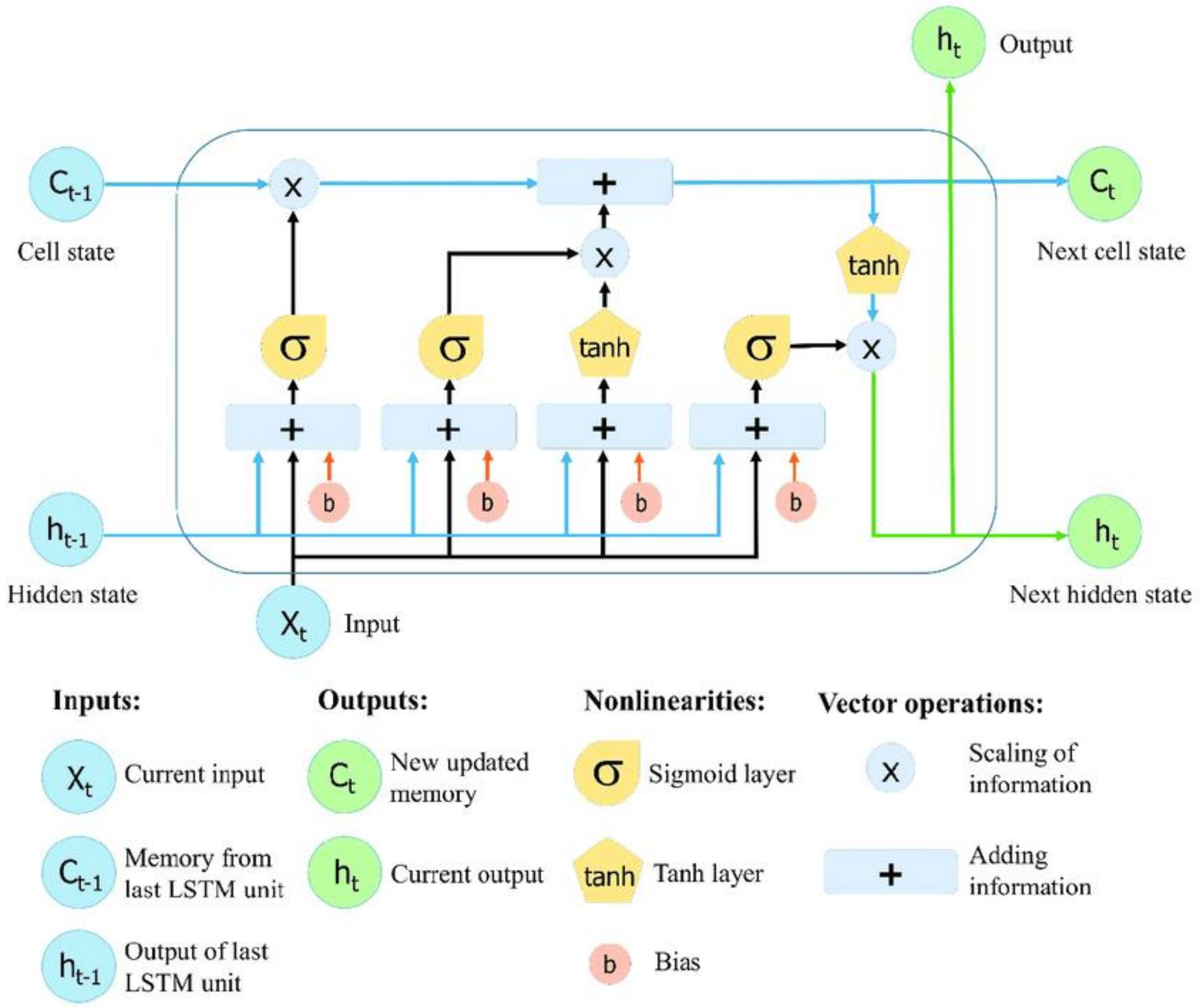

Generative Adversial Network (GAN) models:

GAN is mainly used for generating synthetic data from the original data which helps in improving the model performance. The basic GAN architecture as shown in Figure. 3(A) consists of two main parts: the Generator and the Discriminator. The Generator generates the synthetic samples by taking N-dimensional input variables and the discriminator is a simple classifier that evaluates and distinguishes the generated sample from original samples. There are two losses (generator loss and discriminator loss) that are involved during this process and the entire model is formulated as a minimax game in which the discriminator tries to minimize its reward and the generator tries to minimize or maximize its loss.

Mathematically, GAN model can be expressed as:

$$\frac{min}{G}\frac{max}{D}\mathrm{V(D,G)}$$

$$\mathrm{V(D,G)} = \varepsilon_{x\sim pdata(x)}[logD(x)] + \varepsilon_{z\sim pz(z)}[log(1-D(G(z))] \text{ - - Eq 1}$$

Where, G is Generator, D is Discriminator, pdata(x) is distribution of real data, P(z) is distribution of generator, x is sample from pdata(x), z is sample from P(z), D(x) is the discriminator network and G(z) is the generator network.

Seizure details

S_ID	Seizure file	Thalamic channel	Sampling rate (Hz)	Baseline Duration (s)	Ictal Duration (s)
P7	Event1	FT2-FT3	2048	550	34
	Event2	FT2-FT3	2048	550	46
	Event3	FT3-FT4	2048	550	34
	Event4	FT2-FT3	2048	550	18
	Event5	FT2-FT3	2048	550	24
	Event6	FT2-FT3	2048	550	18
	Event7	FT2-FT3	2048	550	21
	Event8	FT2-FT3	2048	550	17
	Event9	FT2-FT3	2048	550	21
	Event11	FT2-FT3	2048	550	23
P8	Event2	LINS1-LINS2	2048	550	60

	Event5	LINS1-LINS2	2048	550	60
	Event7	LINS1-LINS2	2048	550	58
	Event9	LINS1-LINS2	2048	550	55
	Event15	LINS1-LINS2	2048	550	46
	Event16	LINS1-LINS2	2048	550	65
	Event18	LINS1-LINS2	2048	550	43
P9	Sz1	LT1-LT2	2048	550	158
	Sz13	LT1-LT2	2048	550	93
	Sz14	LT2-LT3	2048	550	106
	Sz16	LT1-LT2	2048	550	70
	Sz17	LT1-LT2	2048	550	99
	Sz18	LT1-LT2	2048	550	97
	Event28	LT2-LT3	2048	550	72
	Event 37	LT2-LT3	2048	550	95
	Event 38	LT2-LT3	2048	550	47
P10	Sz1	RT1-RT2	2048	550	68
	Sz2	RT1-RT2	2048	550	53
	Sz4	RT1-RT2	2048	550	75
	Event 7	RT1-RT2	2048	550	68
	Event 9	RT1-RT2	2048	550	80
	Event 10	RT1-RT2	2048	550	67
	Event 11	RT1-RT2	2048	550	84
P14	Event0	INS2-INS3	2048	550	135
	Sz1	INS3-INS4	2048	550	137
	Sz2	INS3-INS4	2048	550	114
	Sz7	INS3-INS4	2048	550	116
	Sz11	INS1-INS2	2048	550	295
	Sz12	INS1-INS2	2048	550	299
	Sz13	INS1-INS2	2048	550	300
	Sz14	INS1-INS2	2048	550	272
P15	Sz1	RT3-RT4	2048	550	95
	Sz8	RT1-RT2	2048	550	95
	Sz9	RT1-RT2	2048	550	56
	Sz10	RT3-RT4	2048	550	54
	Sz11	RT1-RT2	2048	550	88
	Sz12	RT1-RT2	2048	550	290
P16	Sz1	RT2-RT3	2048	550	125
	Sz2	RT2-RT3	2048	550	43
	Sz3	RT2-RT3	2048	550	55

	Sz4	RT2-RT3	2048	550	112
	Sz5	RT2-RT3	2048	550	32
	Sz6	RT2-RT3	2048	550	41
	Sz7	RT1-RT2	2048	550	100
	Sz8	RT2-RT3	2048	550	76
	Sz9	RT1-RT2	2048	550	246
	Sz10	RT2-RT3	2048	550	125
	Sz12	RT2-RT3	2048	550	30
P17	Sz3	RT2-RT3	2048	550	19
	Sz4	RT2-RT3	2048	550	15
	Sz6	RT2-RT3	2048	550	55
P18	Event 2	RTH3-RTH4	2048	550	238
	Event 3	RTH2-RTH3	2048	550	282
	Event 4	RTH3-RTH4	2048	550	302
P19	Sz2	LT3-LT4	2048	550	54
	Sz3	LT2-LT3	2048	550	75
	Sz4	LT2-LT3	2048	550	110
	Sz5	LT3-LT4	2048	550	102
	Sz6	LT3-LT4	2048	550	108
P20	Sz2	LT3-LT4	2048	550	117
	Sz3	LT3-LT4	2048	550	289
	Sz4	LT2-LT3	2048	550	103
	Sz5	LT2-LT3	2048	550	116
P21	Sz1	RT1-RT2	2048	550	73
	Sz2	RT1-RT2	2048	550	59
	Sz3	RT1-RT2	2048	550	53
	Sz4	RT1-RT2	2048	550	171
	Sz5	RT1-RT2	2048	550	56
	Sz6	RT1-RT2	2048	550	161
P22	Sz1	RT1-RT2	2048	550	136
	Sz2	RT1-RT2	2048	550	107
	Sz3	RT1-RT2	2048	550	116
	Sz4	RT1-RT2	2048	550	126
	Sz5	RT1-RT2	2048	550	133
